# Nurturing gut-brain research: an interview with Helen Vuong on the maternal microbiome in neurodevelopment

**DOI:** 10.1038/s42003-021-02301-z

**Published:** 2021-06-17

## Abstract

Helen Vuong is a postdoctoral fellow in the Hsiao lab at the University of California, Los Angeles, where she is currently funded by a K99 Pathway to Independence Award from the National Institutes of Health. In this Q&A, Dr. Vuong tells us about her current work and the importance of tailoring scientific educational experiences to students. Dr. Vuong also shares tips on how to better support young parents in STEM.

**Figure:**
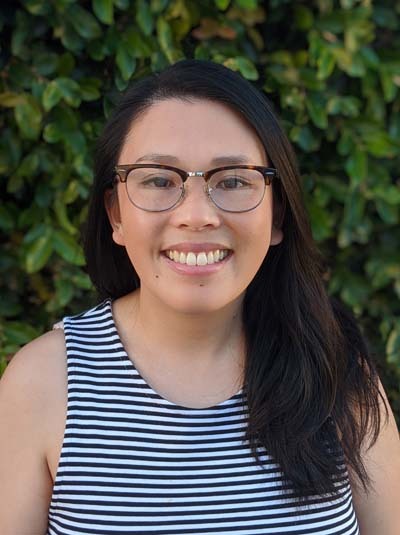
Helen E. Vuong

Please tell us about your research interests.

I am interested in understanding how external factors like the microbiome regulate development and function of the mammalian nervous system. Some questions that I am interested in pursuing for my independent research program are how does the maternal and early-life microbiome contribute to neurodevelopmental processes, how do risk factors for neurodevelopmental disorders like genetics, infection or stress interact with the microbiome to drive changes in brain development, and how do early changes in neurodevelopment have long-term neurosensory, social and cognitive consequences. While my projects will focus on the molecular and electrophysiological mechanisms of neurodevelopment and neurodevelopmental disorders using mouse models, I look forward to forming collaborations with clinicians to facilitate the translation of key preclinical findings.

What initially drew you to the microbiome, after pursuing a PhD in neuroscience? Are there any challenges or risks to switching fields?

My graduate work was with Dr. Nicholas Brecha and Dr. Steven Barnes at the University of California, Los Angeles (UCLA). There I studied the morphological and electrophysiological properties of retinal cells, and how these cells communicated with each other to facilitate vision. In my last year of graduate school, Dr. Elaine Hsiao, who was at Caltech at the time, came to UCLA to give a talk. She presented some fascinating findings where she found that in a maternal infection model the offspring had gastrointestinal barrier disturbances, microbiome alterations and autism-related behavioral abnormalities. When she treated these offspring with a particular bacterium, she could correct the gastrointestinal barrier defects and behaviors. I left the talk completely inspired and so curious to learn “how”. How could the seemingly distant gut bacteria affect central nervous system function?

When I asked Elaine to join her lab, I was caught up in the excitement of doing microbiome research—but I had no idea what that meant. There were inherent challenges like learning the core techniques and grasping the literature. I felt like I was starting graduate school all over again. Although there was a steep learning curve, I found that my graduate training really prepared me for how to think critically, ask a lot of questions, persevere when experiments didn’t work, and just stay curious. Taking those skills combined with a supportive and patient mentor I was able to eventually reach a level of competency. I think that with research you never stop learning, reading and thinking. This underlying curiosity is what ultimately makes you a scientist.

In addition to your scientific successes, you have also been a participant in the UCLA IRACDA teaching-research fellowship. Why is scientific education important to you, and do you have any advice for ECRs to better support their trainees or junior colleagues, especially those who belong to underrepresented minorities in STEM?

The IRACDA program is an NIH K12 career development award. It aims to provide the resources to train scientists to be researchers and teachers in academia. The program is a combined traditional mentored postdoctoral research experience with an opportunity to develop academic skills including teaching through workshops and mentored teaching assignments at a partner institution. My experience with this program has been phenomenal. I have gained life-long mentors and colleagues that are dedicated to motivating and providing the skills for the next generation of students, particularly from underrepresented groups, in the biomedical research fields to become scientists.

As an undergraduate I was naïve to the many science career options. I had thought that a science career meant being a medical doctor, dentist, or pharmacist, until I was lucky enough to join a lab as a dishwasher and was given an opportunity to do some PCR and immunohistochemical staining experiments. The experience opened my eyes to scientific thinking and data collection that could utilize my technical aptitude and sense of curiosity. I realized that maybe the way that science was traditionally taught may not be geared towards students of my same background. To better support students from diverse or underrepresented backgrounds we should not teach or mentor students with a one-size-fits-all strategy, rather we should approach teaching and mentorship as a constantly evolving process. As we continuously educate ourselves with the most effective pedagogy that involves student participation in the thought process, we can also assess and provide feedback on student progress taking into account didactics, career goals, and mental health. I think scientific education has been going through some very meaningful changes. I am really excited to be a part of a new wave of biomedical educators.

Tell us about your journey in research after becoming a parent. What do you think needs to change to better support young parents to continue science?

Being a parent is hard. Being a parent and doing research is an added layer of difficulty. I think that much of research or being successful in research was not designed for parents. There is no “right” time to have a child, and if you want to have a biological child there is a clock that women have to consider. I had my son in my 4th year as a postdoctoral fellow. My experience from having my son, and intensified by the COVID-19 pandemic, revealed to me that there is much to be done in research academia to better support young parents if we want to retain excellent scientists. I think that we need to normalize and have support systems for scientists to have children at any stage of their research careers. I think that it’s important that we have free or heavily subsidized childcare. We also need to mandate lactation rooms in every building. Finally, I think that there needs to be a safe space for women to deal with issues of fertility and postpartum moods. I definitely have a bigger appreciation for my parents and all parents after becoming one.

What do you think will be the next breakthrough in the gut-brain axis?

I think that since the gut-brain axis is a relatively new field, every new discovery is a breakthrough. I personally am looking forward to studies that provide specific cellular and molecular mechanisms for how microbes can interact with brain cells. I am also excited about studies that will use bioengineering to re-engineer a bacterium’s genome to synthesize specific molecules that can have therapeutic implications for neurological disorders.

What is your favorite lab tradition, and do you think you would carry it over when you start your own research group?

There are a few lab traditions that I will carry over to my own research group. Dr. Hsiao organizes amazing lab gatherings once every academic quarter. We’ve done everything from bowling to bubble ball soccer to escape rooms. She also has a tradition of creating congratulatory email threads for anytime undergraduates, graduate students and postdocs wins an award or grant, or when a publication is accepted. Finally, she administers annual lab surveys that assess the state of the lab—areas that are working well and what needs improvement. Ultimately, I want to continue these traditions because they promote a healthy scientific environment that is team-based and constantly evolving to suit the needs of current members.

*This interview was conducted by Associate Editor George Inglis*.

